# What drives local currency bond market development in Saudi Arabia: do macroeconomic and institutional factors matter?

**DOI:** 10.1186/s43093-021-00110-8

**Published:** 2021-12-11

**Authors:** Jamel Boukhatem

**Affiliations:** 1 Deanship of Scientific Research, Umm Al Qura University, Mecca, Saudi Arabia; 2 Faculty of Economic Sciences and Management, University of Tunis El Manar, Tunis, Tunisia

**Keywords:** Bond market development, Macroeconomic and financial factors, Institutional framework, ARDL bounds test, C22, D53, E44, G15, O16

## Abstract

The main objective of this study is to empirically determine which factors are related to the development of local-currency bond market (LCBM) in Saudi Arabia over the period 1990–2019. Using ARDL modeling, the results reveal long-run cointegrating relationships between LCBM capitalization and macroeconomic, financial, and institutional factors. Unlike institutional ones, macroeconomic and financial factors seem to matter more in developing LCBM in the short run. However, in the long run, larger economic size more government spending, low inflation levels, broader and deeper banking system, higher bureaucratic quality, and better investment profile, all play a crucial role in the determination of Saudi LCBM. Policy implications include measures toward sound macroeconomic fundamentals, broad and deep banking system, efficient stock market, and high-quality governance institutions.

## Introduction

Over the last 20 years several international initiatives have been launched to develop local currency bond markets (LCBMs) in developing, emerging, and advanced economies, mainly after the Asian financial crisis of 1997–1998 (G8 action plan 2007, G20 action plan 2011). Subsequently, developing their bond markets has become, for many countries, a high priority and an ultimate goal to achieve. Theoretically, well-developed bond markets are important for at least five reasons. The first one consists at increasing the efficiency and the competitiveness of the financial system which dominated by banks in emerging economies. The second is related to the improvement in financial system stability by creating alternative sources of bank financing reducing consequently their relative power and the related asymmetric-information problems [1–3]. The third one concerns the edification of a veritable yield curve. The fourth reason relates to monetary policy conduct. In fact, deep and liquid government bond markets (GBMs) are essential for the smooth transmission of monetary policy. With well-developed GBMs, central banks can rely on open market operations in conducting monetary policy [4], and acquire valuable information about market reactions to monetary policy moves [5]. Finally, the fifth reason deals with the currency and maturity mismatches. The question of “original sin”—the inability of emerging markets to borrow internationally in their own currency—has emerged with the absence of well-developed LCBMs and the excessive reliance on foreign debt increasing vulnerability in the face of a crisis. The theoretical literature initiated by Eichengreen et al. [6] and then developed by Goldstein and Turner [7] and Ma and Remolona [8], underlines the need to develop domestic bond markets to overcome the problem of double mismatch.

These reasons, among many others, led policymakers in Emerging Asia Economies (EAE) to accelerate the development of their bond markets. They need to promote a broad spectrum of financing instruments and reduce the hegemony of banking system by addressing maturity mismatch issues between the sources of financing and by developing vital local currency-denominated bond markets which have become a necessity to avoid the problems encountered during the crisis and to prepare their financial systems withstanding to potential future shocks. The Asian Bond Markets Initiative (ABMI) started in 2003 has as principal objective the development of efficient and liquid Asian bond markets that would provide a reliable source of long-term finance to the corporate sector. Since the endorsement of this initiative, regional and LCBMs have developed quickly and achieved remarkable growth in terms of size and diversity of issuers, despite the fact that during the peak of the subprime crisis (2007–2009), the quasi-major EAE have known sharp domestic bond outflows.

As the growth of a country’s bond market improves the resilience of its financial system, policymakers in the Emerging East Asia (EEA) region have become more and more interested with the development of strong and viable LCBMs to act as a “spare tire”^1^ in cushioning not only a contraction in credit but also the effect of financial distress on the macro-economy. From another side, in the Middle East and North Africa (MENA) region, bond markets are generally less developed than other emerging Asia markets and particularly than EEA ones. Many factors explain the weakness of MENA bond markets. The structure of demand and supply is the most important among others. Therefore, the demand side is highly dominated by banks while the supply side is government-dominated (except for some Gulf Cooperation Council (GCC) countries), creating a symbiotic relationship between banks and the government strengthened by a lack of other alternative investment projects.

As for Saudi Arabia, it has a sound banking system and a hastily emerging equity market, but the bond market remains immature and underdeveloped. The doing business report of the World Bank and the global competitiveness report of the World Economic Forum show that the access to long-term financing was considered among the main problematic factors for investment in Saudi Arabia, and that financial market development is, relatively, one of the Saudi Arabia’s weaknesses. From another side, a prominent program has launched attempting to develop the Saudi’s bond market by reducing fees and trading commissions for local currency bond transactions. This is an action; but developing debt markets requires determining the appropriate macroeconomic and/or legal and institutional policies.

The Empirical literature shows the extraordinary amount of research conducted on the determinants of the development of stock markets and banking systems. However, limited attention has been ported to bond markets development. The main studies [10, 11], [12–25], [26, 27], Huong [28], etc.) emphasize the importance of a set of fundamental prerequisites in the emergence of well-functioning bond markets. These prerequisites consist of sustained macroeconomic stability and financial soundness, and strong governance and institutional framework.

Unfortunately, while the determinants of bond market development (BMD) in panel data econometrics have been explored widely in emerging and/or advanced countries, little is known about the factors affecting GBMs in country level. Better still, no work has been reserved to the empirical determinants of Saudi bond market development. The main goal of this research consists at studying empirically the role of macroeconomic, financial, and institutional factors in explaining the development of Saudi GBM. In fact, the most prominent program launched by the Saudi Council of Economic and Development Affairs and designed to realize the vision 2030 is the financial sector development program (FSDP). This program aims at creating a dynamic and diversified financial sector with an advanced financial market, in order to boost the national economy.

According to the Saudi Arabia’s 2030 vision, the Kingdom could double its GDP in 2030. To reach this objective, the current economic model based on oil and public spending must give way to a more market-based approach. Moreover, Saudi Arabia has a sound banking system and a hastily emerging equity market, but the bond market remains immature and underdeveloped. The doing business report (World Bank) and the global competitiveness report (World Economic Forum) show that the access to long-term financing was considered among the main problematic factors for investment in Saudi Arabia, and that financial market development is, relatively, one of the Saudi Arabia’s weaknesses.

This paper contributes to the existing literature in several ways. To our body of knowledge, we are the first to focus on the empirical determinants of Saudi GBM development. Our analysis thus reveals how macroeconomic, financial, and institutional factors determine the degree of development of Saudi GBM. Moreover, we estimate an autoregressive distributed lag (ARDL) model of a set of endogenous variables and a vector of exogenous variables describing, respectively, the size of Saudi GBM and the main factors likely to contribute to its development. So, the main contribution of our study is twofold. Taking into account all the factors likely to contribute to the development of Saudi GBM, and using ARDL approach to confirm the long-run and short-run relationships between these factors and the size of GBM where cointegration exists.

The rest of the paper is organized as follows. Section 2 provides a review of relevant theoretical and empirical literature. Section 3 describes data and presents empirical methodology. Section 4 discusses empirical findings. Finally, Sect. 5 concludes the paper.

## Literature review

The literature on the determinants of financial markets development is at the same time quite small and relatively recent. The related studies are beginning to appear in the late 1990s and early 2000s. While these studies address “financial markets”, closer examinations show that rather than covering all the compartments of financial markets they focused really on equity markets. Bond markets are almost completely omitted [27–35]. This omission is due to the small sizes of bond markets compared to equity markets and banking systems in emerging and/or developing economies.

Since recently, researches into the factors leading to more developed emerging bond markets have shown an increasing interest. This is due essentially to the diverse benefits from deep and resilient bond markets. This question has gained further importance after the Asian crisis 1997–1998 and LCBMs are key requirements strengthening the resilience of financial sectors and thereby reducing countries’ vulnerabilities to eventual future crises. But studies that emerged just after these crises are technical rather than theoretical and/or empirical [36–42].

More recent theoretical and empirical studies show that the factors contributing to further developing bond markets are diverse and differ from one economy to another. This is not surprising as long as the size of bond (financial) market depends mainly on the size of the economy. Nevertheless, despite this diversity, we can regroup all these factors in three groups: (macro) economic, financial, and institutional factors.

Notwithstanding the growing body of literature that emerged examining the determinants of BMD, this paragraph is not intended to provide an exhaustive review of the theoretical and/or empirical related literature. In this regard, the main studies examining the factors affecting the size of LCBMs in different regions are: Claessens et al. [43], Eichengreen and Luengnaruemitchai [11, 44], Luengnaruemitchai and Ong [45], Eichengreen et al. [46], Borensztein et al. [47], Burger and Warnock [48], Adelegan and Radzewicz-Bak [49], Garcia-Kilroy and Silva [50], Bhattacharyay [15], Felman et al. [18], Gray et al. [19], Burger et al. [16], Ayala et al. [13], Khalid and Rajaguru [51], and Ahwireng-Obeng and Ahwireng-Obeng [52].

On a panel of 40 countries over the 1990–2001 period, Eichengreen and Luengnaruemitchai [11] explored the determinants of bond market capitalization. They show that larger countries have more capitalized bond markets. These scale or size effects reflect the fixed costs of building appropriate infrastructure such as settlement and clearing systems and reliable legal frameworks for securities issuances and transactions. Such an infrastructure can also be important for the liquidity of secondary markets. In addition, the authors conclude that sound and competitive banking systems are associated with more capitalized bond markets. This constitutes an argument to the fact that financial intermediation (banks) and market intermediation (bonds) are complements rather than substitutes. Finally, effective and strong institutions and stable exchange rates encourage foreign participation in bond markets by minimizing foreign exchange risk.

The studies of Eichengreen et al. [46] and Borensztein et al. [47] are in-line with that of Eichengreen and Luengnaruemitchai [11]. The authors examine the determinants of BMD especially in Latin America and East Asia. However, unlike previous studies, the authors have focused on the determinants of development of the different compartments of these markets (government bonds, corporate bonds) rather than considering the overall bond market capitalization (BMC). The main results show firstly a weakness of the corporate bond markets in the two regions. Secondly, institutional environment and country characteristics accounts for about 70% of the difference in BMC between Latin America and developed countries. Secondly, 25% of the difference in BMC between industrialized countries and Latin America and Asia is due to country size and economic development level. Also, almost 15% of the difference is due to the degree of development of financial system and another 15% is related to historical and geographical factors. The only policy variables that appear to play an important role are macroeconomic stability, trade openness and investor protection, but these may explain at most 25% of the difference between Latin America and East Asia and industrial countries. Policy variables such capital controls, exchange rate regimes, public debt levels, and banking concentration are statistically significant but play only a marginal role in explaining the differences in BMD between Latin America and developed countries.

In the same context, Burger and Warnock [48] distinguish total, government, and private bonds in local-currency bond markets. The results support the negative relationship between inflation variability and bond issues. However, a strong and positive relationship exists between rule of law (creditor rights) and local-currency bond markets capitalization. Finally, a robust fiscal policy ensuring a strong fiscal balance is associated with more local-currency government bond issuance but does not affect local-currency corporate issuance.

Claessens et al. [43] examine the size and currency composition determinants of GBMs for a set of developed and developing countries (local-currency-denominated versus foreign-currency-denominated bonds). The authors show that the increasing number of financial crises can be attributed to the high weight of foreign currencies in government debt markets.

In-line with previous studies, the authors find that macroeconomic and institutional factors (country size, banking system quality, inflation, rule of law, and democracy) affect both local- and foreign-currency bond markets. Also, the exchange rate regime seems to have different effects on domestic- and foreign-currency issuances. Countries with more flexible, de facto or de jure, exchange rates have larger LCBMs but smaller foreign-currency ones. This suggests that pegged exchange rates encourage governments to issue more debt in foreign currencies in order to take into account the advantage of short-term reductions in the cost of the debt service. However, the authors conclude the existence of a crowding-out effect of local-currency issues by foreign-currency ones.

In a relatively more recent study, Burger and Warnock [48] examine the benefits of attracting foreign investors to local bond markets in both developing and developed countries. The authors analyze in which extent countries can attract international investors to their local debt markets. They also mention that the absence of such foreign investors makes countries run the currency mismatches risks, and thereby painful crises. On the other hand, Burger and Warnock [48] point out that foreign participation in US Treasury bond markets has kept interest rates at low levels despite low savings rates. They suggest that countries can enhance foreign participation by enforcing the rule of law and reducing macroeconomic volatility.

The study of Adelegan and Radzewicz-Bak [49] consists empirically at analyzing the determinants of BMD in a panel of 23 sub-Saharan African countries over the period 1990–2008. The major findings of the study are threefold. First, a confluence of factors explains the underdevelopment of domestic bond markets in the region. The most important are the savings constraint, the low level of banking financial intermediation, the small size of banking sector, the structure of the economy, and the high level of corruption. Second, public debt execs a crowding-out effect on private debt. Third, efforts must be directed toward strengthening the investment environment and establishing a regional approach in developing sub-Saharan African bond markets.

Garcia-Kilroy and Silva [50] provide a descriptive analysis of the main characteristics of some selected MENA government bond markets with a minimum size and a greater developing potential (Egypt, Jordan, Lebanon, Morocco, and Tunisia). The authors examine the degree of development of these markets, highlight their main blockages, and propose some catalyzing reforms in order to ensure their development. The results show the existence of several weaknesses explaining the underdevelopment of the above cited markets.

Felman et al. [18] and Gray et al. [19], through a descriptive analysis of financial structure, identify the factors having led to more developed bond markets in ASEAN-5 countries^2^ after the Asian crisis of 1997. It seems that more diversified investor base and more sophisticated technical infrastructure investment are likely to improve the participation of foreign investors. Moreover, sound and competitive banking system reducing the prime lending rates much more gradually and partly than the decline in bond interest rates encourages firms to turn to bond markets.

In the same context of emerging Asia, Burger et al. [16] highlight the factors affecting the size of bond markets especially in smaller economies of the region. The main findings of the study show the role played by high inflation volatility as a veritable impediment to BMD. Moreover, pro-creditor policies and institutions and strong borrowers’ legal rights can enable developing bond markets.

The study of Ayala et al. [13] aims to identify the domestic and global factors having metamorphose the corporate bond markets in 43 emerging economies over the period 2002–2013. The paper focus on the recent boom of some emerging markets. The results show that strong institutions and macroeconomic fundamentals have played an important role in developing bond markets. However, their relative role remains less important during the post-crisis period where investor’s focus shifts toward market size. Global cyclical factors explain therefore most of the changes in the emerging bond markets.

Teplova and Sokolova [25] analyze the tendencies and the determinants of BMD over the period 2006–2015. The authors consider a panel of 15 countries with a vector of macroeconomic and institutional explanatory variables. The results show that local currency devaluation, inflation instability, and exchange rate volatility have significant effects on the size of LCBMs.

The work of Smaoui et al. [23] aims at identifying the key empirical factors of BMD in 22 emerging economies from different regions (Africa, Asia, Latin America, and Eastern Europe) over the period 1990–2013. The empirical results show that combining macroeconomic, institutional, financial, and structural factors lead to more sizeable bond markets. In fact, larger and deep bond markets seem to be associated with greater economic size, more open economies, higher bureaucratic quality, better investment profile, more developed banking sector, and more flexible exchange rate regime. So, developing their economies and following stable macroeconomic policies are the main policy recommendations for countries looking for the development of their bond markets.

Besides, Suriani et al. [26] empirically investigate the existence of long-term cointegrating relationships between macroeconomic determinants and BMD in Indonesia over the period 01:2012–11:2017. The findings show that bond market is significantly affected by the exchange rate, the interest rates, and price levels, while sukuk market is fundamentally affected by the exchange rates.

Finally, Huong [28] examined the factors affecting the development of GBMs in Asean + 3 countries. Based on ordinary least square (OLS), fixed effect, and random effects regressions, the results show the positive effect of economic size, stage of development, and financial integration degree on the size of GBMs. However, the budget balance and the interest rate spread have significant negative influence on the development of GBMs. Besides, no significant relationships have been found between exchange rate variability, banking size and GBMs size.

## Methods

### Data sources and variables description

The empirical literature on the determinants of BMD is unusual, and contains only a handful of statistically significant determinants derived from diverse studies on different regions. These factors are: size of the economy, exchange rate volatility, interest rate variability, inflation, fiscal balance, openness of the economy, size of the banking system, and institutional quality.

In this paper, we empirically analyze the main determinants of the Saudi LCBM using annual observations over the period 1990–2019. We try to cover all types of factors which theoretically are likely to affect the developmental stage of bond markets.

The data on LCBM come essentially from Global Financial Development Database (GFDD), while data on macroeconomic and financial environment come from the World Development Indicators (WDI) and the GFDD of the World Bank. Finally, data on institutional indicators come from International Country Risk Guide (ICRG) database. The dependent variable is the size of the LCBM. This size is measured by the ratio of the amount of outstanding domestic bonds as a share of GDP. This measure covers public domestic debt securities issued by government as a share of GDP.

According to the above literature review, the determinants of BMD can be classified into historical, structural, macroeconomic, financial, and institutional factors. In our analysis, we classify these factors under three subgroups: macroeconomic factors, financial factors, and institutional factors. The set of macroeconomic factors included in the model includes different variables measuring the size of the economy, developmental stage of the economy, trade openness, inflation rate, and government consumption expenditure. However, financial factors include those related to both banking sector and stock market size and efficiency; that is, bank credits to GDP, banking concentration, stock market turnover ratio, and stock market capitalization to GDP ratio. Finally, governance and institutional quality factors include control of corruption index, investment profile index, government effectiveness index, and rule of law index.

According to ICRG definitions, the control of corruption (COR) indicator captures how well each country controls corruption in the form of exploiting political power by government officials for personal gain. The indicator of investment profile (IP) relates to the capacity of a government to create laws and implement efficient policies that encourage private sector development. The government effectiveness (GOV) indicator, however, evaluates the quality of civil and public services, their degree of independence from political pressure, and their implementation, measuring at the end the credibility of government policies. Lastly, the rule of law (RL) indicator reflects “the degree to which citizens of a country are willing to accept the established institutions to make and implement laws and adjudicate disputes”. In other words, the rule of law determines “the extent of protection and enforcement of legal rights of local companies and physical persons” [53]. Given its broad definition in the ICRG, RL index can also be interpreted as a measure of government credibility or rule obedience [54]. Note that since focus is on local currency bonds, the exchange rate variable is not included as foreign bonds are more sensitive to exchange rates changes. Other institutional factors, such as political stability and the absence of violence and voice and accountability, are not included in the model due to data limitations. Table 1 summarizes the variables definitions and their statistical sources as well as their acronyms.

**Table 1:** Variables and data sources

	Variable	Acronym	Description	Exp. sign	Source
Bond market development (BMD)	Public bond market capitalization to GDP	GBMC	Total amount of domestic public debt securities issued (amount outstanding) in domestic market as a share of GDP	na	GFDD
Macroeconomic determinants	Size of the economy	GDP	Real GDP at PPP	+	WDI
	Development stage of the economy: GDP per capita	GDPC	Real GDP per capita at PPP	+	WDI
	Trade openness	EXP	Exports of goods and services as a share of GDP	+ / −	WDI
	Inflation	INF	Percentage change in consumer price index	−	WDI
	Government consumption expenditure	GGFC	General government final consumption expenditure (% of GDP)	+ / −	WDI
Financial determinants	Bank credits to GDP	CRED	Credits to private sector by commercial banks to GDP	+ / −	WDI
	Banking concentration	CONC	Assets of three largest banks as a share of assets of all commercial banks (Herfindhal Concentration index)	−	GFDD
	Stock market efficiency (stock market turnover ratio)	SMTR	Total value of shares traded during the period divided by the average market capitalization for the period	+	GFDD
	Stock market size (stock market capitalization to GDP)	SMC	Value of listed shares to GDP	+	GFDD
Governance and institutional quality determinants	Corruption control	COR	Corruption perception index	+	ICRG
	Investment profile	IP	Investment Profile index	+	ICRG
	Government effectiveness	GOV	Government effectiveness index	+	ICRG
	Rule of law	RL	Rule of law index	+	ICRG

*Source*: the author

### Model specification and empirical methodology

While the question of the factors determining for the development of stock markets has gained considerable attention in academic discussions, there is little empirical work on the determinants of BMD mainly in emerging economies. We use an autoregressive distributed lag (ARDL) “bound test” approach of Pesaran et al. [55] to analyze the long-run relationship between BMD and their different determinants in a multivariate framework. The ARDL specification is appropriate to simultaneously overcome both serial correlation and endogeneity problem among variables [56].

The econometric form of the models relating to GBMD and their macroeconomic, financial, and institutional determinants, once stationarity or cointegration are verified, are as follows:

1 $${\text{Model }}1:GBMC_{t} = \alpha + \beta MAC_{t} + \varepsilon_{t} ,
where MAC = \left( {GDP, GDPC, EXP, INF, GGFC} \right)^{\prime}$$

2 $${\text{Model }}2:GBMC_{t} = \delta + \gamma FIN_{t} + u_{t} , 
where FIN = \left( {CRED, CONC, SMTR, SMC} \right)^{\prime}$$

3 $${\text{Model }}3:GBMC_{t} = \rho + \theta INST_{t} + v_{3t} ,
where INS = \left( {GOV, IP, COR, RL} \right)^{\prime}$$

where $GBMC$ is the Government Bond Market capitalization relative to GDP. $MAC$ and $FIN$ are two matrixes of macroeconomic and financial factors made up of the variables described in Table 1 above. The $INS$ variables are measures of governance and institutional quality and include measures of governance effectiveness, investment profile, corruption control, and rule of law. $\varepsilon_{t}$ is the usual white noise.

Although the ARDL bounds testing approach does not require testing unit root for variables, it is then essential to perform the stationarity properties of each variable in order to avoid the risk of invalid estimation and meaningless results (for I(2) variables), allowing the extraction of short- and long-run parameters. Augmented Dickey–Fuller (ADF) and Phillips–Perron (PP) unit root tests are used for testing variables stationarity. The bound test approach is mainly based on an estimate of the unrestricted error correction model (ECM) using OLS estimation technique. The bound testing approach to cointegration involves investigating the presence of a long-run equilibrium relationship using the ECM frameworks. To investigate the long-run relationships among the variables relating to Eqs. 1, 2, and 3, the ARDL bounds test for the cointegration can be specified as follows (Eqs. 1a, 2a, and 3a):

Equation 1: Model (1)

1a $$\begin{aligned} & \Delta GBMC_{t} = \alpha_{0} + \alpha_{1} GBMC_{t - 1} + \alpha_{2} GDP_{t - 1} + \alpha_{3} GDPC_{t - 1} + \alpha_{4} GGFC_{t - 1} + \alpha_{5} EXP_{t - 1} + \\ & \alpha_{6} INF_{t - 1} + \mathop \sum \limits_{i}^{p} \gamma_{i} \Delta GBMC_{t - i} + \mathop \sum \limits_{j}^{q} \varphi_{j} \Delta GDP_{t - j} + \mathop \sum \limits_{k}^{q} \delta_{k} \Delta GDPC_{t - k} + \mathop \sum \limits_{l}^{q} \lambda_{l} \Delta GGFC_{t - l} + \\ & \mathop \sum \limits_{m}^{q} \eta_{m} \Delta EXP_{t - m} + \mathop \sum \limits_{n}^{q} \mu_{n} \Delta INF_{t - n} + \varepsilon_{t} \\ \end{aligned}$$

Equation 2**: **Model (2)

2a $$\begin{aligned} & \Delta GBMC_{t} = \alpha_{0} + \alpha_{1} GBMC_{t - 1} + \alpha_{2} CRED_{t - 1} + \alpha_{3} CONC_{t - 1} + \alpha_{4} SMC_{t - 1} + \alpha_{5} SMTR_{t - 1} + \\ & \mathop \sum \limits_{i}^{p} \varphi_{i} \Delta GBMC_{t - i} + \mathop \sum \limits_{j}^{q} \gamma_{j} \Delta CRED_{t - j} + \mathop \sum \limits_{k}^{q} \lambda_{k} \Delta CONC_{t - k} + \mathop \sum \limits_{l}^{q} \delta_{l} \Delta SMC_{t - l} + \\ & \mathop \sum \limits_{m}^{q} \mu_{m} \Delta SMTR_{t - m} + u_{t} \\ \end{aligned}$$

Equation 3: Model (3)

3a $$\begin{aligned} & \Delta GBMC_{t} = \alpha_{0} + \alpha_{1} GBMC_{t - 1} + \alpha_{2} GOV_{t - 1} + \alpha_{3} IP_{t - 1} + \alpha_{4} COR_{t - 1} + \alpha_{5} RL_{t - 1} + \\ & \mathop \sum \limits_{i}^{p} \theta_{i} \Delta GBMC_{t - i} + \mathop \sum \limits_{j}^{q} \psi_{j} \Delta GOV_{t - j} + \mathop \sum \limits_{k}^{q} \phi_{k} \Delta IP_{t - k} + \mathop \sum \limits_{l}^{q} \mu_{l} \Delta COR_{t - l} + \\ & \mathop \sum \limits_{m}^{q} \lambda_{m} \Delta RL_{t - m} + \upsilon_{t} \\ \end{aligned}$$

where $\varepsilon_{t}$, $u_{t}$, and $\upsilon_{t}$ are error terms that are white noise, and $\Delta$ represents the difference operator. Conclusions about the long-run cointegration relationships of the variables are made using the bound test F-statistic value.^3^

After confirming the existence of cointegrating relationship among the variables, the second step involves estimating the long-run coefficients of the ARDL model through the following Eqs. (1b, 2b, and 3b):

1b $$\begin{aligned} & \Delta GBMC_{t} = \mathop \sum \limits_{i}^{p} \theta_{i} \Delta GBMC_{t - i} + \mathop \sum \limits_{j}^{q} \delta_{j} \Delta GDP_{t - j} + \mathop \sum \limits_{k}^{q} \lambda_{k} \Delta GDPC_{t - k} + \\ & \mathop \sum \limits_{l}^{q} \mu_{l} \Delta GGFC_{t - l} + \mathop \sum \limits_{m}^{q} \rho_{m} \Delta EXP_{t - m} + \mathop \sum \limits_{n}^{q} \psi_{n} \Delta INF_{t - n} + \varepsilon_{t} \\ \end{aligned}$$

2b $$\begin{aligned} & \Delta GBMC_{t} = \mathop \sum \limits_{i}^{p} \mu_{i} \Delta GBMC_{t - i} + \mathop \sum \limits_{j}^{q} \eta_{j} \Delta CRED_{t - j} + \\ & \mathop \sum \limits_{k}^{q} \delta_{k} \Delta CONC_{t - k} + \mathop \sum \limits_{l}^{q} \gamma_{l} \Delta SMC_{t - l} + \mathop \sum \limits_{m}^{q} \theta_{m} \Delta SMTR_{t - m} + u_{t} \\ \end{aligned}$$

3b $$\begin{aligned} & \Delta GBMC_{t} = \mathop \sum \limits_{i}^{p} \gamma_{i} \Delta GBMC_{t - i} + \mathop \sum \limits_{j}^{q} \varphi_{j} \Delta GOV_{t - j} + \\ & \mathop \sum \limits_{k}^{q} \psi_{k} \Delta IP_{t - k} + \mathop \sum \limits_{l}^{q} \phi_{l} \Delta COR_{t - l} + \mathop \sum \limits_{m}^{q} \omega_{m} \Delta RL_{t - m} + \upsilon_{t} \\ \end{aligned}$$

In this process, we use Akaike information criterion (AIC) to select the appropriate lag length of the ARDL models for all the variables. Finally, short-run dynamics can be estimated through the error correction model (ECM) (1c, 2c, and 3c):

1c $$\begin{aligned} & \Delta GBMC_{t} = \mathop \sum \limits_{i}^{p} \theta_{i} \Delta GBMC_{t - i} + \mathop \sum \limits_{j}^{q} \delta_{j} \Delta GDP_{t - j} + \mathop \sum \limits_{k}^{q} \lambda_{k} \Delta GDPC_{t - k} + \\ & \mathop \sum \limits_{l}^{q} \mu_{l} \Delta GGFC_{t - l} + \mathop \sum \limits_{m}^{q} \rho_{m} \Delta EXP_{t - m} + \mathop \sum \limits_{n}^{q} \sigma_{n} \Delta INF_{t - n} + \psi ECT_{t - 1} + \varepsilon_{t} \\ \end{aligned}$$

2c $$\begin{aligned} & \Delta GBMC_{t} = \mathop \sum \limits_{i}^{p} \mu_{i} \Delta GBMC_{t - i} + \mathop \sum \limits_{j}^{q} \eta_{j} \Delta CRED_{t - j} + \mathop \sum \limits_{k}^{q} \delta_{k} \Delta CONC_{t - k} + \\ & \mathop \sum \limits_{l}^{q} \gamma_{l} \Delta SMC_{t - l} + \mathop \sum \limits_{m}^{q} \theta_{m} \Delta SMTR_{t - m} + \psi ECT_{t - 1} + u_{t} \\ \end{aligned}$$

3c $$\begin{aligned} & \Delta GBMC_{t} = \mathop \sum \limits_{i}^{p} \gamma_{i} \Delta GBMC_{t - i} + \mathop \sum \limits_{j}^{q} \varphi_{j} \Delta GOV_{t - j} + \\ & \mathop \sum \limits_{k}^{q} \tau_{k} \Delta IP_{t - k} + \mathop \sum \limits_{l}^{q} \phi_{l} \Delta COR_{t - l} + \mathop \sum \limits_{m}^{q} \omega_{m} \Delta RL_{t - m} + \psi ECT_{t - 1} + \upsilon_{t} \\ \end{aligned}$$

## Results and discussion

**Fig. 1:**
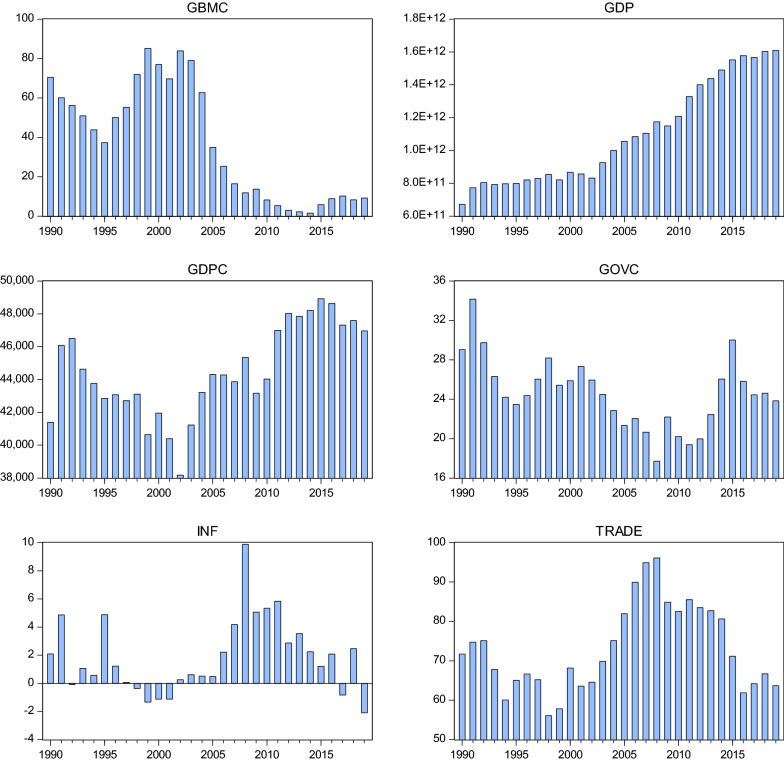
Graphical representation of the variables of model 1

**Fig. 2:**
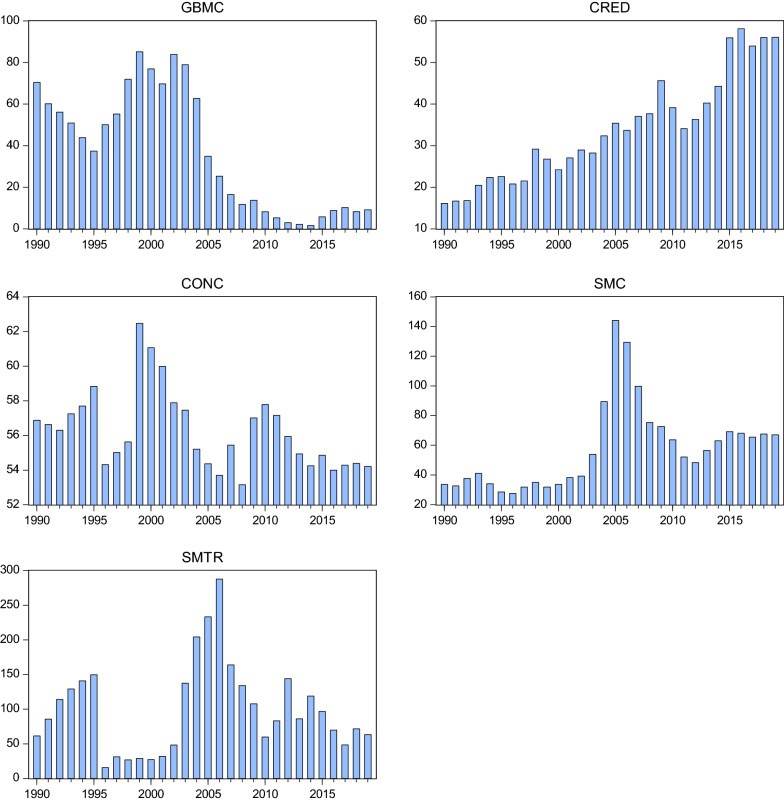
Graphical representation of the variables of model 2

**Fig. 3:**
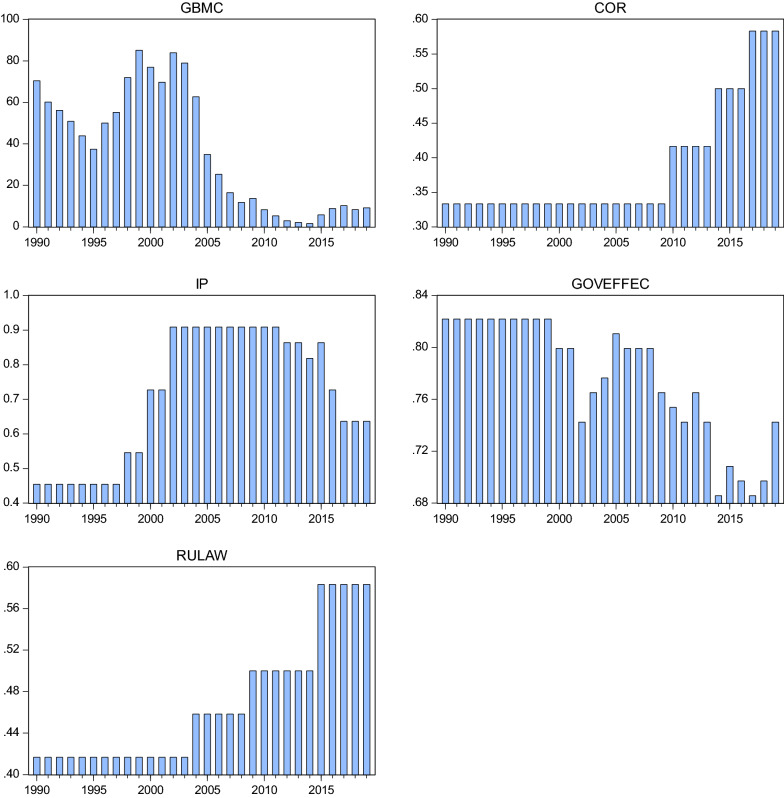
Graphical representations of the variable of model 3

This section presents the empirical results from different test statistics as well as their corresponding interpretations. Before that, the graphical representation of the different groups of variables corresponding, respectively, to the three models exist in appendix (Figs. 1, 2, and 3). To properly interpret ARDL models results, we follow Yacouba and Altıntaş [57, 58], Yacouba et al. [59], and Altıntaş and Yacouba [60].

### Unit root test results

Before testing of cointegration, we apply the augmented Dickey–Fuller (ADF) test and the nonparametric Phillips–Perron (PP) test to determine the stationarity of variables and their order of integration. The tests will involve the three options “none,” “constant,” and “constant and trend”; only statistically significant results are reported here.

As we can see from Table 2, the results of the ADF test on level illustrate that all series are nonstationary at level, and stationary at first difference and are consequently integrated of order one I(1), except for inflation which is stationary at level (I(0)). The same results are found when applying the PP test strongly supporting that all data series are stationary after the first difference except for inflation which is I(0). Hence, these results demonstrate that, rather than the Johansen cointegration model, the ARDL model is appropriate in analyzing the data,^4^ to investigate the impact of macroeconomic, financial, and institutional factors on the Saudi government BMD.

**Table 2:** Results of ADF and PP unit root tests

	ADF test statistic		PP test statistic		Order of integration
	Level	First difference	Level	First difference	
$GBMC$	− 2.243 (0.449)	− 3.256 (0.027)	− 1.480 (0.127)	− 3.256 (0.002)	I(1)
$GDP$	3.983 (0.999)	− 4.293 (0.000)	3.855 (0.999)	− 4.316 (0.000)	I(1)
$GDPC$	− 1.749 (0.396)	− 5.908 (0.000)	− 1.825 (0.361)	− 5.908 (0.000)	I(1)
$GGFC$	− 2.204 (0.209)	− 5.662 (0.000)	− 2.145 (0.229)	− 5.662 (0.000)	I(1)
$EXP$	− 1.401 (0.839)	− 4.416 (0.008)	− 1.543 (0.790)	− 4.618 (0.005)	I(1)
$INF$	− 2.236 (0.026)	− 8.240 (0.000)	− 2.104 (0.036)	− 8.127 (0.000)	I(0)
$CRED$	− 3.223 (0.100)	− 4.721 (0.004)	− 2.711 (0.239)	− 7.844 (0.000)	I(1)
$CONC$	− 2.324 (0.171)	− 3.541 (0.014)	− 2.350 (0.164)	− 10.818 (0.000)	I(1)
$SMC$	− 3.004 (0.148)	− 3.890 (0.026)	− 0.510 (0.486)	− 2.785 (0.007)	I(1)
$SMTR$	− 2.243 (0.196)	− 3.221 (0.029)	− 2.311 (0.175)	− 4.917 (0.000)	I(1)
$GOV$	− 2.682 (0.250)	− 3.334 (0.082)	− 2.780 (0.215)	− 5.027 (0.001)	I(1)
$IP$	0.724 (0.230)	− 2.003 (0.045)	0.049 (0.690)	− 5.147 (0.000)	I(1)
$COR$	− 0.539 (0.975)	− 6.417 (0.000)	− 1.000 (0.928)	− 7.672 (0.000)	I(1)
$RL$	− 2.021 (0.564)	− 4.472 (0.007)	− 1.925 (0.615)	− 12.855 (0.000)	I(1)

The values in (.) are the p-values

### Cointegration test results

Before running the cointegration test, we estimate VAR Model for selecting the optimum lag order. Based on Akaike information criterion (AIC), the optimal lags suggested are 2, 1, and 2 for the models 1, 2, and 3, respectively (Table 3).

**Table 3:** VAR models for optimal lag selection

Lag	LR	FPE	AIC	SC	HQ
$${\text{Model}}\;1:GBMC, GDP, GDPC, EXP, INF, GGFC$$					
0	NA	118.541	7.605	7.893	7.691
1	18.085	51.914	6.775	7.111	6.875
2	4.277*	44.913*	6.623*	7.008*	6.738*
3	0.208	48.200	6.686	7.118	6.815
$$Model\;2: GBMC, CRED, CONC, SMC, SMTR$$					
0	NA	443.007	8.927	9.167	8.998
1	45.418*	55.041*	6.838*	7.126*	6.924*
2	0.068	59.339	6.909	7.245	7.008
3	2.695	55.796	6.841	7.225	6.955
$$Model \, 3:GBMC, GOV, IP, COR, RL$$					
0	NA	437.023	8.913	9.153	8.984
1	34.938*	89.437	7.323	7.611	7.409
2	3.049	83.074*	7.245*	7.581*	7.345*
3	0.037	89.842	7.317	7.701	7.431

After selecting the optimum lag order, we estimate the Eqs. 1a, 2a, and 3a using the OLS method. The calculated F-statistics for the cointegration test for the three models are displayed in Table 4.

**Table 4:** Results of the Bounds test of Cointegration

*Estimated Model (1)*	$F_{GBMC} \left( {GBMC/GDP, GDPC,GGFC, EXP, INF, } \right)$			
$$Optimal \;Lag \;Length\; \left( {AIC} \right)$$	(2,2,0,1,0,2)			
$F - Statistics \left( {Bound \;Test} \right)$ ^a^	4.383^*^			
$Critical\; Values$	1%	2.5%	5%	10%
$Lower \;Bounds\; I\;\left( 0 \right)$	3.06	2.7	2.39	2.08
$Upper\; Bounds\; I\;\left( 1 \right)$	4.15	3.73	3.38	3.00
$R - squared$	0.979			
$Adj.\; R-squared$	0.962			
$DW$	2.665			
$F-Statistics$	59.196^*^			
*Estimated Model (2)*	$F_{GBMC} \left( {GBMC/ CRED, CONC, SMC, SMTR} \right)$			
$$Optimal \;Lag\; Length\; \left( {AIC} \right)$$	(1,0,1,0,0)			
$F - Statistics \;\left( {Bound\; Test} \right)$ ^a^	5.899^*^			
$Critical \;Values$	1%	2.5%	5%	10%
$Lower \;Bounds\; I\;\left( 0 \right)$	3.29	2.88	2.56	2.2
$Upper \;Bounds\; I\;\left( 1 \right)$	4.37	3.87	3.49	3.09
$R - squared$	0.960			
$Adj. R-squared$	0.949			
$DW$	2.023			
$F - Statistics$	88.721^*^			
*Estimated Model (3)*	$F_{GBMC} \left( {GBMC/ GOV, IP, COR, RL} \right)$			
$$Optimal\; Lag Length\; \left( {AIC} \right)$$	(2,0,2,0,0)			
$F - Statistics\; \left( {Bound \;Test} \right)$ ^a^	3.58			
$Critical \;Values$	1%	2.5%	5%	10%
$Lower \;Bounds \;I\left( 0 \right)$	3.74	3.25	2.86	2.45
$Upper\; Bounds\; I\left( 1 \right)$	5.06	4.49	4.01	3.52
$R-squared$	0.959			
$Adj. \;R-squared$	0.942			
$DW$	2.120			
$F-Statistics$	56.669^*^			

^*^ and ^***^ represent significance levels at 1% and 10%. The critical values mentioned in the above table were obtained from Pesaran et al. [55]. ^a^ The ARDL models are performed with restricted intercept and no trend option (Case 2). DW represents Durbin–Watson test statistics

The decision rule is based on comparing F-statistic to lower and upper bounds. If the F-statistic is higher than the upper bound value, we reject the null hypothesis of no cointegration. Consequently, long-run cointegration relationships exist and ARDL can apply. However, the null hypothesis is accepted when the F-statistic is lower than the lower critical bound; no long-run relationship can exist and ARDL could not be used. If the F-statistic value falls between the lower and upper boundaries, then the test is indecisive.

According to the results of Table 4, we found that there is cointegration relationship between macroeconomic factors and GBMC (model 1), financial factors and GBMC (model 2), and institutional factors and GBMC (model 3) at 5%, 1%, and 10%, respectively.

### Short-run and long-run estimated coefficients

The results of the estimated short-run and long-run ARDL models for the period 1990–2019 are reported in Tables 5 and 6 below. The optimal lag-lengths indicated by the AIC for the three models are (2,2,0,1,0,2), (1,0,1,0,0), and (2,0,2,0,0), respectively.

**Table 5:** Estimated short-run coefficients

$\mathrm{Variable}$	$\mathrm{Coefficient}$	$\mathrm{t}-\mathrm{statistics}$	$\mathrm{Prob}.$
*Model (1): dependent variable: ΔGBMC*			
$\Delta GDP$	− 0.254	− 0.856	0.933
$\Delta GDP\left( { - 1} \right)$	0.288	0.728	0.943
$\Delta GDP\left( { - 2} \right)$	− 0.158	− 0.576	0.577
$\Delta GDPC$	0.002	2.747**	0.049
$\Delta GDPC\left( { - 1} \right)$	− 0.002	− 0.251	0.806
$\Delta GDPC\left( { - 2} \right)$	0.003	0.519	0.614
$\Delta GGFC$	1.003	2.412**	0.024
$\Delta GGFC\left( { - 1} \right)$	1.025	1.092	0.297
$\Delta GGFC\left( { - 2} \right)$	1.109	1.549	0.149
$\Delta EXP$	− 0.367	− 0.774	0.455
$\Delta EXP\left( { - 1} \right)$	− 0.278	− 0.585	0.569
$\Delta EXP\left( { - 2} \right)$	− 0.058	− 0.116	0.909
$\Delta INF$	− 0.103	− 2.598**	0.047
$\Delta INF\left( { - 1} \right)$	0.835	0.665	0.520
$\Delta INF\left( { - 2} \right)$	0.132	0.156	0.878
$Constant$	27.081	3.731*	0.002
$ECT_{t - 1}$	− 0.939	− 4.408*	0.001
$F - statistic$	1.593		0.029
$R - squared$	0.718		
$DW$	1.605		
*Model (2): dependent variable: ΔGBMC*			
$\Delta CRED$	0.328	0.664	0.515
$\Delta CRED\left( { - 1} \right)$	0.038	0.079	0.937
$\Delta CONC$	− 0.410	− 0.404	0.690
$\Delta CONC\left( { - 1} \right)$	− 0.849	− 0.867	0.397
$\Delta SMC$	− 0.296	− 1.967***	0.065
$\Delta SMC\left( { - 1} \right)$	0.074	0.483	0.634
$\Delta SMTR$	− 0.058	− 1.356	0.192
$\Delta SMTR\left( { - 1} \right)$	− 0.022	− 0.518	0.610
$Constant$	− 1.139	− 0.639	0.530
$ECT_{t - 1}$	− 0.380	− 4.059*	0.001
$F - statistic$	2.035		0.094
$R - squared$	0.544		
$DW$	1.871		
*Model (3): dependent variable: ΔGBMC*			
$\Delta GOV$	− 0.267	− 1.991***	0.071
$\Delta GOV\left( { - 1} \right)$	− 0.230	− 0.938	0.368
$\Delta GOV\left( { - 2} \right)$	− 0.275	− 1.308	0.217
$\Delta IP$	0.231	0.355	0.728
$\Delta IP\left( { - 1} \right)$	− 0.628	− 0.980	0.347
$\Delta IP\left( { - 2} \right)$	− 0.110	− 1.646	0.127
$\Delta COR$	− 0.110	− 0.648	0.530
$\Delta COR\left( { - 1} \right)$	− 0.227	− 1.175	0.264
$\Delta COR\left( { - 2} \right)$	1.484	1.185	0.267
$\Delta RL$	− 0.227	− 0.141	0.890
$\Delta RL\left( { - 1} \right)$	− 0.161	− 1.175	0.264
$\Delta RL\left( { - 2} \right)$	0.221	1.088	0.299
$Constant$	− 0.455	− 0.125	0.902
$ECT_{t - 1}$	− 0.186	− 2.786**	0.019
$F - statistic$	4.026		0.049
$R - squared$	0.583		
$DW$	1.831		

^*^, ** and *** represents significance at 1%, 5%, and 10% level, respectively

**Table 6:** Estimated long-run coefficients

*Model (1): dependent variable: GBMC*			
${\text{Variable}}$	${\text{Coefficient}}$	${\text{t}} - {\text{statistics}}$	${\text{prob}}.$
$GDP$	0.039	3.611*	0.001
$GDPC$	0.004	3.546*	0.001
$GGFC$	1.759	2.427**	0.023
$EXP$	− 0.124	− 0.487	0.630
$INF$	− 2.204	− 2.249**	0.033
$Constant$	2.420	5.734*	0.000
*Model (2): dependent variable: GBMC*			
$CRED$	− 1.755	− 4.614*	0.000
$CONC$	3.703	1.973***	0.059
$SMC$	0.366	1.538	0.136
$SMTR$	− 0.175	− 1.905***	0.068
$Constant$	− 0.115	− 1. 017	0.318
*Model (3): dependent variable: GBMC*			
$GOV$	0.211	2.150**	0.033
$IP$	0.317	2.027**	0.019
$COR$	0.723	0.729	0.576
$RL$	− 0.485	− 0.668	0.510
$Constant$	0.494	2.221**	0.035

^*^, ** and *** represent significance at 1%, 5%, and 10% level, respectively

#### Macroeconomic determinants

For the first model using solely macroeconomic factors (model 1), three among five coefficients are significant in the short run. The first interesting results suggest that the developmental stage of the economy proxied by GDP per capita, government consumption expenditure (GGFC) and inflation rate (INF), constitute important factors having significant impacts on the capitalization of Saudi GBM. The positive sign of the coefficient assigned to GDP per capita suggests that Saudi economy has attained a certain degree of development allowing it to have larger government bond market. These results seem to be in line with the most studies’ findings on the determinants of BMD [62, 11, 21, 51, 52]

Moreover, government consumption expenditure (GGFC) and inflation rate (INF) have expected signs. Inflation (government consumption) has a negative (positive) and statistically significant influence on Saudi GBM at 5% significance level. In fact, an increase (reduction) in government spending (inflation) may lead to an increase in the size of LCBM. This result signifies that high inflationary expectations lead to uncertainty among investors and discourage consequently bond market activities. Saudi Central Bank behavior in containing inflation helped reducing such uncertainties and contributed consequently to developing GBM. This finding is consistent with Burger and Warnock [10], Essers et al. [63], Khalid and Rajaguru [51], and Boukhatem et al. [64].

Finally, the coefficient of trade openness, as measured by total exports to GDP ratio, is not significantly different from zero at the 5% significance level, which implies that it does not appear to play a major role in developing Saudi GBM. This result is not surprising since, in the literature of BMD, the impact of trade openness on Government BMD is not as forthright as is the economy size, and that this could be especially important when studying corporate bond market or the currency composition of bonds.

In the long run, the results are slightly different. So, in addition to GDP per capita, government consumption, and inflation rate which already significantly impacted GBM capitalization in the short run, the size of the economy, as proxied by its GDP, also constitutes another determinant of GBM development. This result intersects with almost all those studying the determinants of bond market development.

#### Financial determinants

The second model (model 2) includes the effects of the variables characterizing the development of different segments of the financial system (banking sector and stock market) on GBM development. The results of the short-run estimations showed that only the coefficient of the variable measuring the size of the stock market is significant. The negative sign of the coefficient assigned to this variable shows the existence of a substitutability relationship between GBM and stock market activity. This result seems to be in line with the Saudi Arabia 2030 vision where structural economic reforms and initiatives have been launched including, among others, the promotion of the private sector and the enhancement of public finance sustainability. This result corroborates those of [44, 62–67]. In the long run, however, the size of the banking sector, as proxied by bank credits to the private sector, is significantly and negatively associated to GBM development. This result confirms that bonds and banks are competing sources of external finance. In Saudi Arabia, the banking system is so sophisticated to succeed in crowding-out bonds from market share.

Moreover, bank concentration has a positive and significant impact on GBM capitalization. This result contradicts with the previous literature stating a negative connection between the two variables [65–70].

Besides, we notice that while the size of the stock market is no longer significantly related to the development of Saudi GBM, its efficiency seems to have a negative and significant effect on BMD. An efficient stock market restrains the development of a LCBM by reducing the cost of equities, causing an increase in risk appetite and thereby reducing the attractiveness of raising debt in the bond market [21, 71].

#### Governance and institutional quality determinants

Model 3 exposes the contribution of institutional peculiarities in developing GBM. The short-run estimation results show that, among four measures of the quality of institutions, only the government effectiveness index has a significant and negative effect on GBM development. However, in the long run, government effectiveness and investment profile indexes contribute positively and significantly to the development of GBM in Saudi Arabia. More developed governance institutions spur thereby bond market development. This result is coherent with the empirical literature that stipulates that well-developed institutions matter for economic and financial development through efficiently allocating resources to their productive uses in the economy [69–74]. Indeed, one percent increase in government effectiveness index brings about 0.211 percentage point increase in bond market capitalization (Table 6). This relationship is expected and concurs with the underlying theory of government effectiveness and bureaucratic quality. A higher government effectiveness index should represent a more stable and efficient country with better quality of public and civil service along with its separation from political pressures, conducting thereby to credible government policies that should be associated ultimately with the increase in the amount outstanding of public domestic debt securities. According to ICRG methodology, the investment profile index “provides an assessment of factors affecting the risk to investment”. The higher the investment profile index, the lower the risk for investors. So, the positive coefficient assigned to this variable suggests that government with strong and effective investment profile can lead to improvement in market returns, thereby arising the bond market capitalization.

Finally, the corresponding error correction coefficients are significantly negative, which further confirms the established cointegration between GBM capitalization and its different macroeconomic, financial, and institutional determinants. The $\left( {ECT_{t - 1} } \right)$ estimates are − 0.455, − 0.359, and − 0.186, respectively, implying that GBMC displaces with 45.5%, 35.9%, and 18.6% adjustment speed from the short-run disequilibrium toward the long-run equilibrium, respectively.

### Results of diagnostic tests

To test the robustness of the estimated models, we applied the LM Breusch–Pagan test for heteroscedasticity, LM Breusch–Godfrey test for autocorrelation, Jarque–Bera normality test, and Ramsey RESET test to validate the correctness of functional form of selected models. The relevant F-stats and Prob ${\chi }^{2}$ are reported in Table 7 below. The results suggest that the computed models were error-free (absence of heteroscedasticity and serial correlation, normal distribution and no functional form misspecification).

**Table 7:** Diagnostic tests for long run

Diagnostic test	LM BP $\chi^{2}$	LM BG $\chi^{2}$	JB $\chi^{2}$	Ramsey Reset Test $\chi^{2}$
Model 1	1.641 (0.187)	1.258 (0.303)	1.447 (0.484)	1.248 (0.243)
Model 2	1.546	0.109	0.009	1.264
	(0.206)	(0.897)	(0.995)	(0.224)
Model 3	0.487 (0.902)	0.621 (0.558)	3.055 (0.217)	0.559 (0.588)

The numbers in the brackets are the *P*-values

**Fig. 4:**
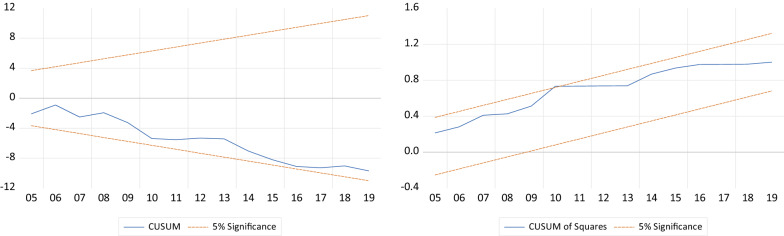
CUSUM and CUSUMSQ long-run analysis stability tests of model 1

**Fig. 5:**
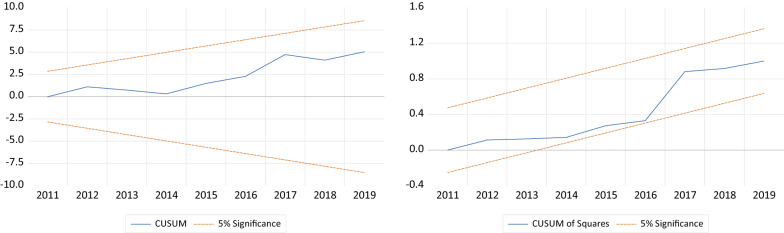
CUSUM and CUSUMSQ long-run analysis stability tests of model 2

**Fig. 6:**
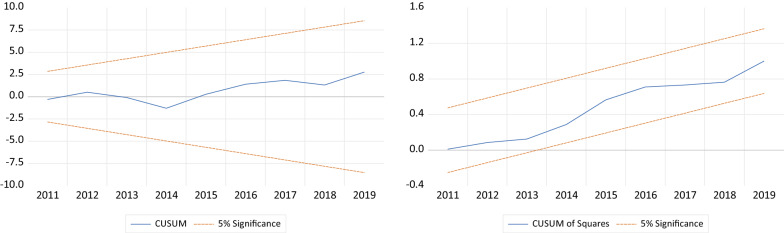
CUSUM and CUSUMSQ long-run analysis stability tests of model 3

Finally, to further validate the stability of the models, we use the cumulative sum (CUSUM) of recursive residuals and the cumulative sum of squares (CUSUMSQ) of recursive residuals. Graphically, in all models the CUSUM and CUSUMSQ plots fall within the boundaries (Figs. 4, 5, and 6 of the appendix), meaning that the null hypothesis of unstable parameters is rejected, and consequently, the ARDL estimates appeared to be stable.

## Conclusion

The prime objective of this study was to scrutinize empirically the ultimate determinants of LCGBM development in Saudi Arabia over the period 1990–2019. It contributes to the existing literature by using an ARDL bounds testing approach to test the contribution of macroeconomic, financial, and institutional factors, respectively, in developing Saudi GBM.

The cointegration test results indicate the existence of long-run cointegrating relationships between GBM capitalization and the aforementioned factors. Empirical findings show that a confluent of macroeconomic, financial, and institutional factors contributes to the development of Saudi GBM in the long run. Indeed, larger and more developed economy, more government spending, low inflation levels, broader and deeper banking system, higher bureaucratic quality, and better investment profile, seem to play crucial roles in the determination of Saudi GBM development.

More recently, Saudi Arabia is looking at domestic and international bond markets to lower fiscal deficits caused last years by falling oil revenues and coronavirus crisis; with the listing of government debt instruments on Saudi stock market, raising of debt is expected to be more liquid and transparent. An efficient and well-functioning GBM could help providing liquidity by broadening the investor base, providing thus financial stability.

Finally, from the above findings this paper provides insightful recommendations for policy makers and competent authorities:

- Policymakers are required to implement appropriate and attractive macroeconomic policies to hold local-currency-denominated debt instruments and thereby develop active and efficient LCGBM.
- Given the important role the banking sector plays in developing GBM, more financial innovations in the banking industry are encouraged in order to improve efficiency, and promote complementarity between bank-based and market-based financing.
- The Saudi Arabia Monetary Authority (SAMA) can use GBM to pursue a targeted monetary policy. Therefore, a good coordination between the monetary authority and the government is essential to reap this benefit and to strengthen the transmission mechanism of monetary policy.
- Finally, the investment environment should be enhanced. Greater efforts would be made to strengthening the reliability of rule of law implementation and reducing bureaucratic practices, therefore raising economic development and ultimately developing Saudi GBM.

## Data Availability

The dataset analysed during the current study is available from the author on reasonable request.

## Appendix

See Figs. 1, 2, 3, 4, 5, and 6 .

## Abbreviations

LCBM: Local currency bond market
GBM: Government bond market
EAE: Emerging Asia Economies
ABMI: Asian bond markets initiative
EEA: Emerging East Asia
MENA: Middle East and North Africa
GCC: Gulf cooperation council
BMD: Bond market development
BMC: Bond market capitalization
GBMC: Government bond market capitalization
FSDP: Financial sector development program
ARDL: Autoregressive distributed lag
OLS: Ordinary least square
GFDD: Global financial development database
WDI: World development indicators
ICRG: International country risk guide
COR: Control of corruption
IP: Investment profile
GOV: Government effectiveness
RL: Rule of law
GDP: Gross domestic product
GDPC: Gross domestic product per capita
UECM: Unrestricted error correction model
ADF: Augmented Dickey–Fuller
PP: Phillips–Perron
EXP: Exports of goods and services
INF: Inflation
GGFC: General government final consumption
CRED: Credits to private sector
CONC: Banking concentration
SMTR: Stock market turnover ratio
SMC: Stock market capitalization
MAC: Macroeconomic variables
FIN: Financial variables
INS: Institutional variables

## Footnotes

1 The “spare tire” role of a well-functioning bond market has been used by Greenspan in a well-known speech [9].

2 Asian-5 comprises Indonesia, Malaysia, Philippines, Singapore, and Thailand.

3 If the F-statistic value is greater (lower) than the upper (lower) critical value bounds, then the variables are (not) cointegrated. Finally, if the F-statistic value is between the upper and the lower critical value bounds, then the decision is inconclusive.

4 The ARDL approach provides consistent results even in an existing mix order of I(0) or I(1), or a mutually integrated order of variables. Moreover, this test is unusually efficient and consistent with small and finite sample sizes [61].
